# Mobile applications for road traffic health and safety in the mirror of the Haddon’s matrix

**DOI:** 10.1186/s12911-021-01578-8

**Published:** 2021-08-02

**Authors:** Hossein Aghayari, Leila R. Kalankesh, Homayoun Sadeghi-Bazargani, Mohammad-Reza Feizi-Derakhshi

**Affiliations:** 1 Department of Health Information Technology, School of Management and Medical Informatics, Tabriz University of Medical Sciences, Tabriz, Iran; 2 Health Services Management Research Center, Research Center of Psychiatry and Behavioral Sciences, Tabriz University of Medical Sciences, Tabriz, Iran; 3 Road Traffic Injury Research Center, Tabriz University of Medical Sciences, Tabriz, Iran; 4 International Safe Community Certifying Center, Stockholm, Sweden; 5 Department of Computer Engineering, University of Tabriz, Tabriz, Iran

**Keywords:** m-Health, Traffic accident, Prevention, Traffic safety, Haddon’s matrix, Public health

## Abstract

**Background:**

Road traffic accidents have been one of the leading causes of death. Despite the increasing trend of road traffic apps, there is no comprehensive analysis of their features and no taxonomy for the apps based on traffic safety theories. This study aimed to explore the characteristics of available mobile apps on road traffic health/safety and classify them with emphasis on Haddon’s matrix.

**Methods:**

The researchers examined the mobile applications related to road traffic health/safety using qualitative content analysis. Google Play was searched using a combination of the keywords. Haddon’s matrix was applied to analyze and classify those mobile apps residing in the categories of Road Traffic health & Safety, and Road Traffic Training.

**Results:**

Overall, 913 mobile apps met the inclusion criteria and were included in the final analysis. Classification of the apps based on their features resulted in 4 categories and 21 subcategories. A total number of 657 mobile apps were classified based on Haddon’s matrix. About 45.67% of these apps were categorized as the road traffic health & safety group.

**Conclusions:**

Haddon’s matrix appears to have the potential to reveal the strengths and weaknesses of existing mobile apps in the road traffic accident domain. Future development of mobile apps in this domain should take into account the existing gap.

**Supplementary Information:**

The online version contains supplementary material available at 10.1186/s12911-021-01578-8.

## Background

According to the World Health Organization report, 1.35 million people are killed due to traffic accidents, and millions are injured annually [1]. Moreover, road accidents have psychological consequences. Based on studies, road traffic accidents have been argued to be the leading cause of posttraumatic stress disorder (PTSD) [2]. As WHO reports on the global status of road safety, despite being among the leading causes of mortality, most traffic accidents are predictable and preventable. There is evidence of interventions that have been effective at preventing traffic accidents; countries that have successfully implemented these interventions have seen a considerable decrease in traffic accident deaths [3].

Considering the scope and pace of technological improvements, people have turned to technologies for solving their various problems [4]. Mobile phones are one of the most widely used technologies. The number of mobile subscribers was 5.2 billion in 2019. It is estimated to have about 5.8 billion unique mobile subscriptions worldwide by the end of 2025 [5].

Smartphone is a culprit for drivers’ distraction and road accidents [6–10]. One study highlighted that tasks in the smartphones that take drivers' eyes off the road have a greater safety–critical risk than functionalities with no involvement of the eyes off the road [11]. However, another study has shown the conversation on hands-free (HF) and handheld (HH) phones can affect the driver reaction time (RT: as a response with a brake pedal to an event requiring a response in the traffic environment), RT detection, detection percentage, lateral position and speed in a similar way. This can be attributed to the fact that if the phone does not fit in the car automatically, interacting with the HF phone may require manipulating a device. Therefore the driver may require keeping his eyes off the road, which may increase the risk of an accident [12].

Despite being among the main culprit behind road traffic accidents, smartphone technology can facilitate road traffic management practices. Different capabilities of smartphones (such as lane detection, vehicle detection, and vehicle distance estimation) can turn them into potential accident prevention devices [13]. With the widespread use of mobile devices, many companies have recently developed apps to improve public service quality, people security, and safety [14]. Similarly, the development of smartphone technologies has driven the growth of mobile applications in the road traffic field. These apps are used for many transportation-related activities such as education, road traffic data collection, travel information, route planning, and navigation [15].

There are also traffic apps for promoting environmental safety, drivers/pedestrian safety, traffic alerts [16], and driving behavior feedbacking. The world has witnessed progress in these areas. For example, receiving traffic accident information via mobile apps has changed for recording driver behavior. According to recent studies, receiving information about driving behavior through traditional models such as questionnaires, Police report studies, and direct observation has been replaced with new approaches, including [17]:

1. Driving simulator: This type of device is designed to imitate driving and enables a safe, virtual environment for testing driver behavior characteristics.
2. Naturalistic driving: This includes installing instruments such as In-Vehicle Data Recorders (IVDRs) and On-Board Diagnostics (OBDs) tools in the vehicle to capture information about driving behavior in the real world.
3. Non-intrusive recording of driver’s behavior: This involves the use of smartphone data captured by sensors embedded in smartphones. One can use such data for both accident analysis and road safety research.

Using smartphone sensors for recording the driving behavior data of 303 drivers at the road segment and junction level showed that if the average traffic volume per lane increases in the respective areas, the number of rough events in the road segments will increase. In addition, as the average occupancy increases in junctions, there is an increase in harsh accelerations, and as the average speed increases, more harsh decelerations occur [18].

As the number of road traffic apps increases, several issues may arise and influence their use. This increase makes identification of the apps difficult, making their classification inevitable. One way to help researchers and professionals to understand and analyze complex domains is to classify objects [16]. According to evidence [19], any taxonomy should have the following characteristics if it is to be beneficial: It must be brief, enough inclusive, comprehensive, and extendible.

A limited body of knowledge is available on the classification of apps relates to general mobile app classification methods such as hierarchical classification, two-dimensional classification, and three-dimensional classification [20]. There is no classification of apps in a particular domain based on their related scientific frameworks. Only two studies in the literature [21, 22] have presented the mobile app classification in traffic and tourism fields. According to one of the studies, road traffic apps [23] fall into the following three types:

1. Blocking apps: relates to apps that prevent or limit the driver from using routine functionalities of the mobile phone, such as calling, typing, reading, and various notifications.
2. Apps that change the interface with the user: include apps that present a less distracting interface by enabling “Eyes on the road hands on the wheel”. This is done through voice controls, heads-up displays (HUDs), and hand gesture control.
3. Driving feedback and coaching apps: are safety-oriented apps that provide, similar to In-Vehicle-Data-Recorder (IVDR), also known as Green Box, indications about unsafe and aggressive behaviors, collision warnings on short headways and lane-keeping, fatigue detection, and unexpected weather conditions.

Since each of the apps has a variety of features, characterizing them is crucial for appropriate use. It is essential to identify the whole spectrum of the apps' functionalities and enlist information and communication media used in mobile apps. The researchers characterized Information Feeding System (IFS) used by the apps. The aim was to enlist technologies of capturing information and types of communication media transferring information to end-users. Smartphones with a high-resolution camera, microphone, compass, accelerometer, 3-axis gyroscope, and GPS sensors can collect different data. They can capture traffic information to detect congestion, traffic signals, and rerouting traffic. These devices can also gather environmental information for monitoring road conditions to detect road anomalies and warn drivers of potholes and their location. Moreover, they can sense information about driving behavior to recognize aggressive or nonaggressive driving, drunk driving, lane departure, and assist eco-driving [21].

Despite the increasing trend of the apps on road traffic health and safety, there is no comprehensive analysis and classification of their features based on the traffic safety frameworks. This study aimed to explore the characteristics of available mobile apps on road traffic health and safety and classify them based on Haddon’s matrix as the most comprehensive framework in the area of injury prevention. As a mobile operating system, Android is widely used and has 72% of the mobile market share [23], compared with other operating systems.

William Haddon had worked for many years on road safety in the USA. In 1970, he presented Haddon’s Matrix to the world of injury prevention [24]. Researchers have used the Haddon matrix as a tool for developing ideas for safety promotion and prevention of various injuries or fatalities. The matrix is a framework table with three rows and four columns related to the public health concepts. This framework aims at changing the concepts of primary, secondary, and tertiary prevention [25]. Although Haddon initially presented his matrix for incident analysis at the individual level, this matrix has been used at the level of group analysis and even in methodological studies as a method of qualitative analysis. In the present study, the Haddon matrix is used as a research methodology to provide more practical study objectives and at the same time improve the level of study analysis and facilitate the output of the analysis for users in the field of traffic safety who are familiar with the Haddon matrix [26]. Each row frames the timing of incident respectively as pre-event, event, and post-event phase. While designing a mobile app for primary prevention, we can categorize it as an intervention under the pre-event phase. For example, the apps that provide road traffic rules education and apps that inform about the vehicle status before the trip belong to this category. Analyzing the event phase will be valuable when interested in secondary prevention measures. For example, apps for drowsiness and distraction management relate to this phase. Apps for driving/driver behavior feedbacking belong to the post-event phase of Haddon’s matrix.

## Methods

**Fig. 1:**
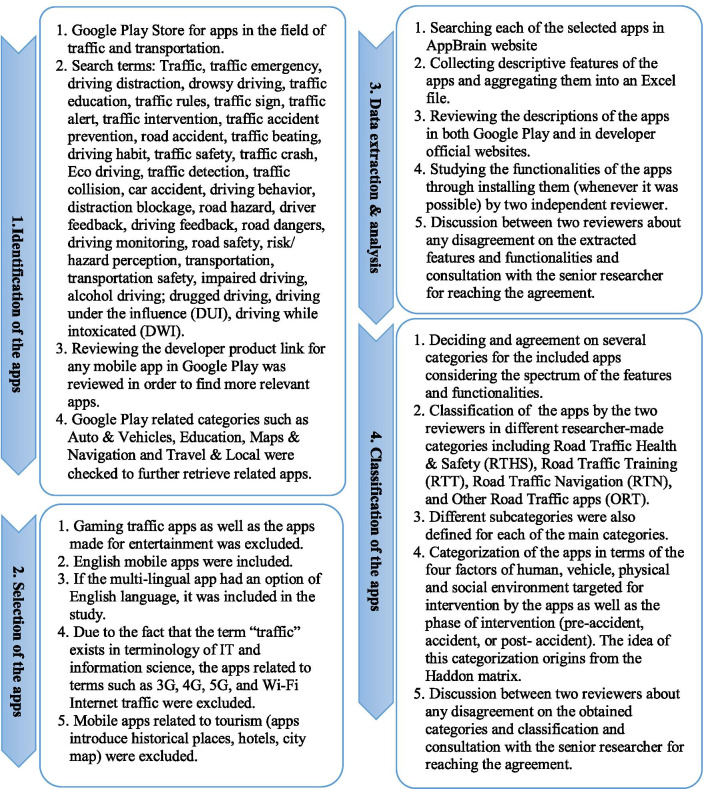
Stages for analysis and classification of the road traffic mobile apps

A comprehensive review of the mobile applications for road traffic health and safety was carried out using qualitative content analysis. This study included all apps related to the field of road traffic. First, the researchers set eligibility criteria to retrieve the most relevant apps in the search phase. After retrieving the apps, the researchers (HA and LRK) screened them for selecting the appropriate apps. Then data was extracted about the apps and their features. In addition, their functionalities were analyzed. Finally, the apps were classified in general and in terms of Haddon’s matrix framework. Figure 1 illustrates these stages in detail.

In this study, we extracted the features of traffic and transportation apps. If they had health and safety attributes, the researchers categorized them using Haddon’s matrix. According to the app features, we created four categories: Road Traffic Health & Safety (RTHS), Road Traffic Training (RTT), Road Traffic Navigation (RTN), and Other Road Traffic Apps (ORT). Out of the four categories, two groups (RTHS and RTT) had health and safety features. Therefore, we divided them based on Haddon’s matrix. We considered Haddon’s matrix factors as the human, vehicle, physical, and social environment. Then we categorized the apps based on these factors. We also modified the phases of Haddon’s matrix to the pre-driving, driving, and post-driving stages. We finally classified the apps based on these phases. The supplementary file [see Additional file 1] includes details of data extracted from the mobile apps in terms of 4 factors and three stages of Haddon’s matrix.

## Results

### Basic information about reviewed apps

**Fig. 2:**
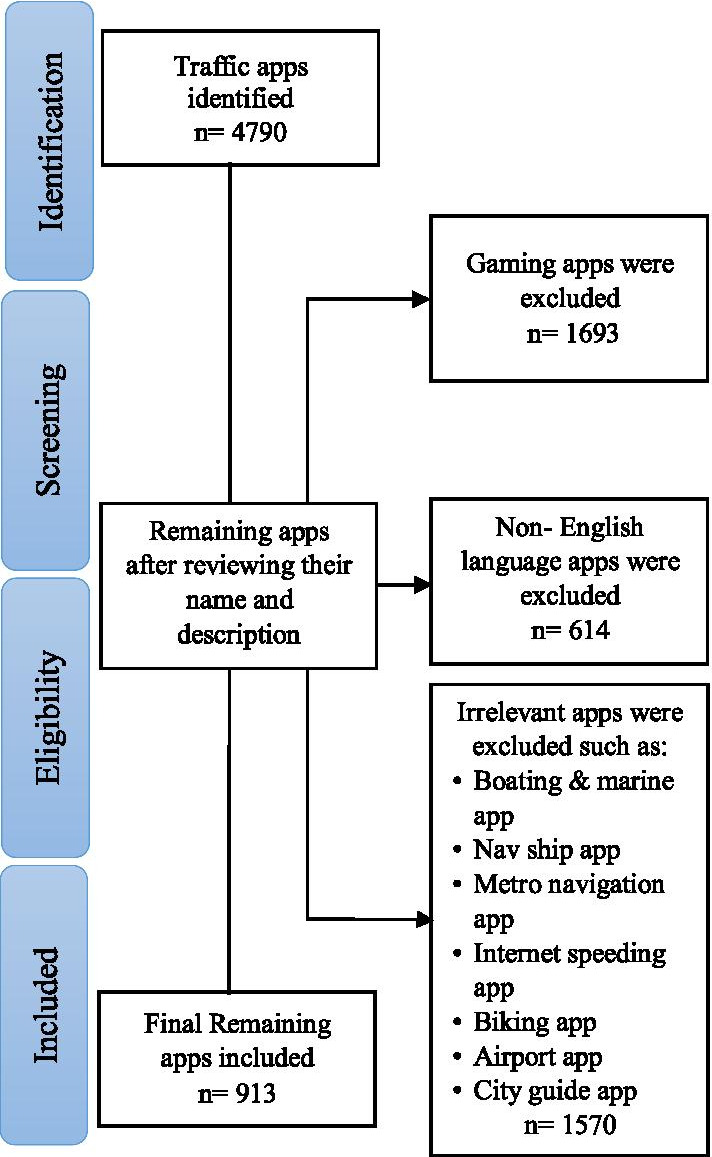
Flow chart of reviewing road traffic mobile apps for representing the process of searching, screening, and selecting

As shown in Fig. 2, a total number of 4790 apps were retrieved from Google Play Store. After reviewing and screening them, 913 apps remained for the final review after applying eligibility criteria. About 82.3% of the reviewed apps were free, 16.9% FREE + In-App, and 0.9% Paid. The launch date of the reviewed apps revealed that the creation date of most apps were 2018 and 2017, with 251 and 190 apps, respectively.

### The general classification of the reviewed apps

**Fig. 3:**
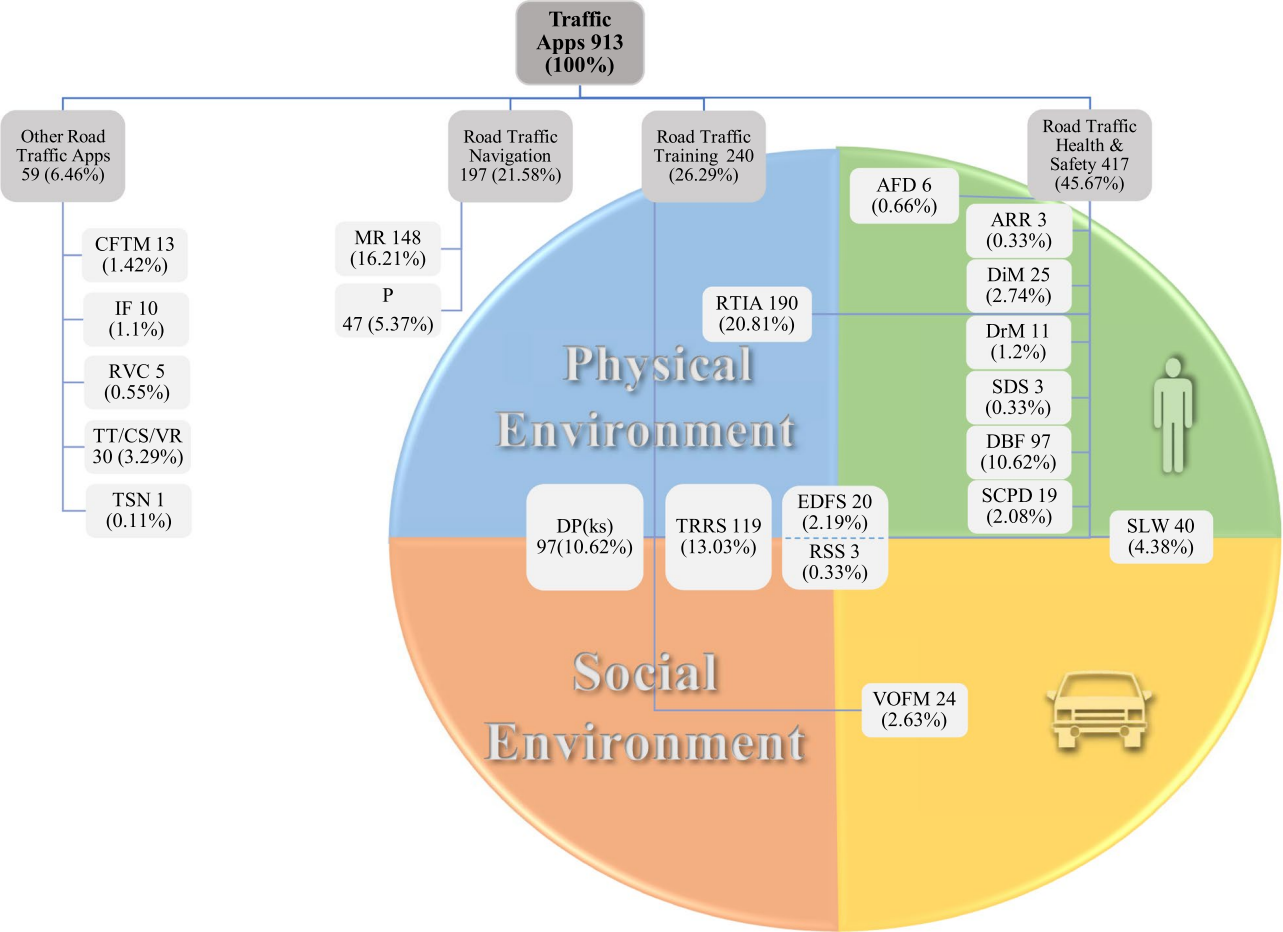
The classification of road traffic apps according to Haddon’s matrix. Road Traffic Health & Safety (RTHS): *ARR* Accident record and report,* AFD *Alcohol free driving, *DrM* Drowsiness management, *DiM* Distraction management, *DBF* Driving/driver behavior and feedbacking, *SDS* Safe driver service, *SCPD* Speed camera & police detector, *SLW* Speed limit warning, *RTIA* Real-time traffic information/ alerting, *EDFS* Eco driving & fuel saving, *RSS* Ride sharing service, Road Traffic Training (RTT): *VOFM* Vehicle operating, fixing and maintenance, *TRRS* Traffic rules & road signs, *DP(ks)* Driving performance (knowledge & skills). Road Traffic Navigation (RTN): *MR* Mapping & routing, *P* Parking. Other Road Traffic apps (ORT): *CFTM* Car or fleet tracking and management, *IF* Insurance & fine, *RVC* Remote vehicle control, *TT/CS/VR* Taking taxi/Car service/Vehicle renting, *TSN* Transportation for special need

To classify the apps, two researchers (HA and LRK) analyzed the app features and functionalities independently, which agreed on 75%. The remaining 25% was discussed by the two reviewers, of which about 8% disagreed, and eventually, disagreements were resolved by the third researcher (HSB). Four general categories with 21 subcategories were defined for the apps (Fig. 3). The four categories include Road Traffic Health & Safety (RTHS), Road Traffic Training (RTT), Road Traffic Navigation (RTN), and Other Road Traffic apps (ORT).

The RTHS category has 11 subcategories. Among the subcategories of the RTHS group, the highest number of apps (190) is the real-time traffic information/alerting apps that monitor road traffic issues such as congestion, road construction, accident, and weather conditions to inform drivers near to real-time or in real-time for preventing potential hazards.

Among 240 apps in the RTT category, 13.03% of apps are in the subcategory of traffic rules & road signs. In addition, apps that help people learn the driving knowledge and skills (with the use of image, video, and tips) to receive a driving license and also apps that distinguish risk perception of the driver are grouped in the driving performance (knowledge & skills) subcategory with 10.62%.

The RTN category consists of 197 apps in 2 subgroups. Apps under mapping & routing subcategories (16.21%) are similar to the apps under the real-time traffic alerting subgroup, except that there are no warnings about road conditions, accidents, and blockage. The priority in this subcategory is to guide users through the route provided on the map or by voice. Moreover, the ridesharing service (0.33%), safe driver service (0.33%), and Eco- driving & fuel saving (2.19%) are categorized in the RTHS group. The apps of these subcategories are classified in the RTHS group because of their health and safety features. For example, Eco-driving & fuel saving subcategory has apps to manage fuel consumption and reduce environmental pollution while managing traffic issues with safety implications. The ridesharing service subcategory has apps to transform urban mobility by providing timely and convenient transportation. It has the potential for a positive impact on society in terms of pollution and energy consumption. Also, the parking subcategory with 5.3% apps allows users to find and book free parking spaces.

The ORT category has five subcategories. Among the subcategories of the ORT group, the highest number of apps (30) is related to the apps intending to take taxi/car service/vehicle renting.

### Classification of the apps by the Haddon’s matrix

Figure 3 illustrates the achieved classification of the road traffic apps based on four factors of human, vehicle, physical and social environment of Haddon’s matrix. Utilizing these factors revealed the feasibility of classifying two main categories of the apps (including the RTT and the RTHS apps) based on Haddon’s matrix. For example, the drowsiness management subcategory involves the human factor of Haddon’s matrix. Also, the speed limit warning subcategory involves both human and vehicle factors of Haddon’s matrix. Furthermore, eco-driving & fuel-saving, and ride-sharing service subcategories involve all 4 of Haddon’s factors.

A total number of 657 apps were categorized into two RTHS and RTT groups. The factor/s that has/have been the center of attention for intervention in apps relates to the physical & social environment (32.88%) in the RTT category, followed by the physical environment (28.92%), human (24.96%), human-vehicle (6.09%), vehicle (3.65%), and all four Haddon’s matrix factors (3.5%).

One hundred percent of the apps residing in the Event/driving phase of Haddon’s matrix belong to the RTHS category. Most of the apps under the Event/driving phase belong to the real-time traffic information/alerting subgroup. These apps perform in real-time while driving. Moreover, 12.41% of the apps under the Pre-event/Pre-driving phase belong to the RTHS group, and 87.59% belong to the RTT group. The traffic rules & road signs subcategory is ranked first (43.43%) among subcategories of apps that belonged to the Pre-driving/Pre-event phase. Most of the apps classified under the Post-event/Post driving phase are related to the driving/driver behavior feedbacking subcategory (96.67%), which informs drivers about their driving behavior at the end of the trip.

Since some of the apps had features targeting more than one phase of Haddon’s matrix, we considered more than one phase when required. For example, some apps of Real-time traffic information/alerting subgroups can only be used by the user before driving (for example, by providing traffic information such as a blocked route, and they notify the driver before starting the trip and moving to that route). However, some of them are also used by the user while driving. Such an app may inform the user of traffic information such as heavy traffic on the road resulting from road construction or an accident in real-time. Moreover, some apps in this subcategory have features for helping before, during, and after driving. Table 1 presents the full details.

**Table 1:** Distribution of RTHS apps as well as RTT apps in Haddon’s matrix

	(Pre-event/ Pre-driving)	(Event/ Driving)	(Post- event/ Post-driving)	(Pre-event/ Pre-driving) & (Event/ Driving)	(Pre-event/ Pre-driving) & (Post- event/ Post-driving)	(Event/Driving) & (Post- event/ Post-driving)	(Pre-event/ Pre-driving) & (Event/ Driving) & (Post- event/ Post-driving)
*Road Traffic Health & Safety (RTHS*)	12.41%	100%	100%	100%		100%	
Accident recording and reporting		1.06%					
Alcohol free driving	2.19%						
Distraction management	0.36%	7.75%				7.41%	
Driving/driver behavior feed backing	0.36%	4.93%	96.67%			88.89%	
Drowsiness management		3.87%					
Eco driving & fuel saving	6.2%		3.33%	8.33%			
Real-time traffic information/ alerting	1.09%	61.62%		91.67%		3.7%	
Ride sharing service	1.09%						
Safe driver service	1.09%						
Speed camera & police detector		6.69%					
Speed limit warning		14.08%					
*Road Traffic Training (RTT)*	87.59%						
Driving knowledge & skills	35.4%						
Traffic rules & road Signs	43.43%						
Vehicle operating,fixing and maintenance	8.76%						
Total	100%	100%	100%	100%		100%	

### Communication media and information feeding systems of the apps

**Fig. 4:**
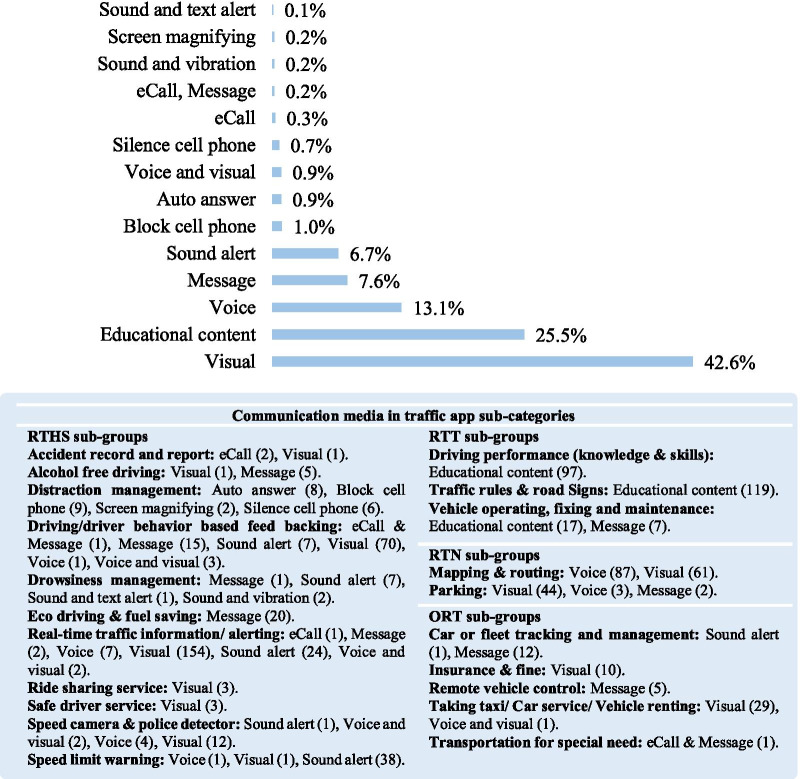
Distribution of communication media in different subcategories of road traffic apps

The researchers characterized fourteen types of communication media considering the features of apps. The auto-answer functionality has been used commonly in apps classified in the subcategory of distraction management. By deploying this technology, the mobile application automatically answers incoming calls with messages like "I'm driving”, “I call later” or others. As Fig. 4 illustrates, visual communication media is the most used technology among mobile apps, so that 42.6% of the apps in all four categories have utilized this technology. Overall, 154 apps from real-time traffic information/alerting subgroups provide users with a visual technology of the traffic situation. 233 apps included educational content technology containing video, text, and picture for the apps with training functionality.

By deep analyzing the features of included apps, nine different types of information feeding sources (IFS) were characterized. These sources were categorized into two groups: static IFS and dynamic IFS. While only off-line IFS is grouped into the category of static, others are classified into the dynamic IFS (see Table 2 for details).

**Table 2:** Distribution of information feeding sources (IFS) among different subcategories of the road traffic apps

Type and combination of IFS	Definitions and examples	Frequency	Distribution of IFS among different subcategories of apps
Mobile sensing	Collecting and analyzing information are done by mobile sensors such as GPS, gyroscope, accelerometer, Bluetooth, and camera	378 (41%)	Accident record and report (3), Alcohol free driving (1), Distraction management (22), Driving/driver behavior based feedbacking (27), Drowsiness management (9), Eco-driving & fuel saving (12), Real-time traffic information/ alerting (16), Ridesharing service (3), Safe driver service (3), Speed camera & police detector (13), Speed limit warning (36), Vehicle operating, fixing and maintenance (2), Mapping & routing (144), Parking (48), Car or fleet tracking and management (5), Remote vehicle control (3), Taking taxi/ car service/ Vehicle renting (30), Transportation for special need (1)
Mobile sensing and earlobe sensor	Driver's fatigue and drowsiness symptoms detecting by ear signals alongside mobile sensing	1 (0%)	Drowsiness management (1)
OBD adaptor	On-Board Diagnostics is a computer-based system designed for monitoring the performance of major engine components. The information captured from the OBD adaptor is sent to the mobile app via the Internet of Things (IoT). Furthermore, the telematics adapter apparatus includes an onboard diagnostics (OBD) port connected to the vehicle for retrieving information about the self-calibrating accelerometer, driver behavior pattern recognition and advanced accident telemetry data (26)	31 (3%)	Distraction management (1), Driving/driver behavior based feedbacking (29), Real-time traffic information/ alerting (1),
Mobile sensing and OBD adaptor	Both OBD device and mobile sensor used for information collecting and analyzing	26 (3%)	Distraction management (2), Driving/driver behavior based feed backing (7), Eco driving & fuel saving (8), Real-time traffic information/ alerting (4), Car or fleet tracking and management (5)
Mobile sensing and community based	Traffic information obtained from both mobile sensors and community users. For example drivers share real-time traffic and road information via their smartphones	69 (8%)	Driving/driver behavior based feed backing (1), Real-time traffic information/ alerting (54), Speed camera & police detector (6), Speed limit warning (3), Mapping & routing (4), Parking (1)
Mobile sensing and traffic camera	Besides mobile sensors, traffic information is obtained from traffic control cameras	115 (13%)	Real-time traffic information/ alerting (114), Speed limit warning (1)
Mobile sensing and valid activation code	These apps should be provided with a valid activation code from apps official websites that are used in driving/ driver behavior based feed backing and car or fleet tracking and management subcategories	36 (4%)	Driving/driver behavior based feed backing (33), Car or fleet tracking and management (3)
Off-line (standalone)	These apps are able to operate without control from another system, or company, and don't need an Internet connection or active GPS for function. After launching the app, the user can use app information	256 (28%)	Alcohol free driving (5), Drowsiness management (1), Driving performance (knowledge & skills) (97), Traffic rules & road Signs (119), Vehicle operating, fixing and maintenance (22), Insurance & fine (10), Remote vehicle control (2)
Traffic radio	Only one app uses traffic radio for information collecting. Traffic Radio 96.1 FM app belongs to Lagos State (Nigeria). The State Government realized the challenges of traffic jams and concluded that heavy traffic congestion on roads should be reduced. Finally a dedicated traffic radio was established to monitor traffic and safety matters in Lagos state	1 (0%)	Real-time traffic information/ alerting (1)

## Discussion

In this study, we first explored the characteristics of available mobile apps on road traffic health and safety and then classified them based on Haddon’s matrix as the most comprehensive framework in the area of injury prevention. Haddon's efforts aimed at using systems theory to explain ways to reduce the frequency and severity of traffic accidents [27]. We could not consider the Haddon framework as a systems theory approach. However, it reflects the importance of three levels of prevention and the importance of working with all elements of the system, not just the road user, to identify causes as well as preventive actions [28].

By classifying the apps in terms of Haddon’s matrix, we were able to find out which categories of apps can help users before, during, and after driving. Also, we found that each of the apps is related to which Haddon’s matrix factors (human, vehicle, social and physical environment) toward reducing the errors of that factor in driving. For instance, if a driver is drowsy during driving, the drowsiness management app can warn him/her at the right time before getting late. As another example, if there is a problem in the driving route (physical agent error), the apps of real-time traffic information/alerting subgroups can help the driver and prevent possible accidents.

Haddon’s matrix provided us a relevant framework for structured analyses of the mobile apps in the domain of Road Traffic Health & Safety. We classified the apps in terms of 3 different factors of Haddon’s matrix (including human, vehicle/equipment, and environmental factors) and three phases of the pre-accident, accident, and post-accident. When we applied Haddon’s matrix factors to different subgroups, we found that the human factor is the most widely considered factor for digital intervention in mobile applications across all the phases, before, during, and after driving.

According to the results, a few apps have mentioned that they have been developed or supported by traffic and transportation organizations, and most of them were individuals or software companies. However, there were relevant organizations among the developers including, the Abu Dhabi Department of Transport (DOT), Keeping Roads Safe Technologies Inc., Cambridge Mobile Telematics, American Geriatrics Society, National Highway Traffic Safety Administration, Wyoming Department of Transportation, Department of Transport, and Main Roads Queensland. Supporting such organizations may reflect a recognition and perceived importance of mobile apps in road traffic health and safety. Since there is no evidence, we could not judge the quality of apps based on their designers or sponsors. However, it appears that the more we could have an engagement of road traffic safety experts and the more we could use the evidence for designing the apps, we may end up with higher quality mobile apps. Such an approach may decrease the individuals' concern regarding the safety of the apps. During the evaluation of the mobile apps, one may ask if there is any road traffic safety organization and evidence behind the developed app.

Since traffic behaviors are more culturally relevant, they should be considered as a strategic and ongoing goal. Moreover, every possible solution should be used for improving traffic safety. One of the technology-based solutions could be the road traffic apps developed, approved, and recommended by the relevant agencies or authorities. It appears imperative to have organizations in place for examining, validating, and accrediting the apps produced, especially if their developers have some claims about road traffic health and safety, and accident prevention.

We also looked at the communication media used in mobile apps. Communication media that was observable among apps under the distraction management subcategory ranged from the auto-answer to Block cell phone, Silence cell phone, and Screen magnifying. Managing communication media in multi-functional apps appears critical since different tasks such as drowsiness management, distraction management, real-time traffic alerting, driving behavior feedback or other features may require different communication media.

The other issue that matters in app development is the information feed. If credible centers feed information into the mobile application, their validity will be more likely. These centers may include and are not limited to traffic control centers, police, road network administration, emergency medical centers, and insurance companies. It appears the reliable information sources can lead to the effective management of road traffic issues and give confidence to the app users. Evidence shows that some of the apps developed in the field of traffic health and safety (ranging from environmental detection [29, 30], to drowsiness management [31, 32], medical supporting [33, 34], real-time road condition [35, 36], and driving behavior [37–39]) provide information feeds using mobile sensors.

Smartphones have the potential to prevent possible accidents. Researchers have developed a mobile app that prevents accidents by monitoring, analyzing driving behavior, and advising driver based on unsafe driving behaviors. They used smartphone cameras and internal sensors (accelerometer, gyroscope, GPS, and microphone) to monitor driving behavior [40]. It is important to note that many mobile apps with the functionality of driving safety have been developed. However, there is limited empirical evidence on whether these apps are effective in promoting road safety or not. Furthermore, the evaluation of these apps through clinical trials seems to be inevitable. The field evaluators should assess the effectiveness, efficiency, and impact of these apps on health and traffic safety for the most popular apps in the real world or simulated environment. Moreover, the validity of these apps should be evaluated. Because they are in road traffic safety and their poor performance may cause irreversible injury or fatal results.

Authorities and legislators should regulate the use of road traffic health and safety apps in terms of their approval, encouragement, or prohibition. They should take a position on the validity and use of these apps. These authorities can also provide some domain standards and red lines for the development of road traffic apps. It appears inevitable to define a clearance process and dedicated clearance body for approval of these apps similar to what we have as FDA clearance for medical apps. This can contribute to the safety and accuracy of the apps. Therefore, the user may use them with confidence and trust.

## Limitations

Our study was limited to the apps developed for android platforms and did not include apps developed for other platforms. In most cases, the retrieved apps on Google Play have an iOS version. Despite the fact that both operating systems have sufficient popularity worldwide, the penetration of the Android operating system is more than iOS. Another limitation of our study is that we did not analyze the user comments for the apps included in this study. The results from such analysis could identify the issues related to the usage of apps in the real world. It could also shed light on the unmet needs of the user and reveal their recommendations for consideration in future app updates or developments.

## Future direction

Future studies should examine and evaluate traffic apps on iOS and other existing operating systems. Moreover, it is also recommended to do original research on existing apps and compare their safety and performance of their features. It is also ideal to evaluate the user-friendly of traffic apps with evidence of secondary data in a simulated environment or real world. Future studies should define a suitable and comprehensive final model for the development and evaluation of traffic apps. Such a model can be used as a reference for developing apps that are more compatible with road traffic safety goals and have full features.

Finally, the following users may use the results from this study:

1. Road users. To get acquainted with the existing road traffic apps.
2. Politicians, legislators, and managers. By studying the features of existing apps, they can develop relevant rules and promote traffic safety-related training. Managers can also increase the awareness of road users about these apps by getting acquainted with the features related to traffic apps.
3. Researchers (Road Safety Researchers; Information Technology Researchers or App Research Scientists). Road safety researchers can evaluate safety aspects and user-friendliness of traffic safety apps in the form of original studies. In addition, the features of apps presented in this study can guide information technology researchers in suggesting appropriate frameworks for the development of road traffic apps for the use of app developers.
4. App developers. By studying the features of existing apps and getting acquainted with their strengths and weaknesses, they can develop a more appropriate, comprehensive, and compatible app for traffic safety purposes.
5. News reporters can present the results of app classification and their features to general target groups.

## Conclusion

Haddon’s matrix proved useful in categorizing existing mobile apps of road traffic health and safety. It means the mobile apps can be designed and used as an intervention on different factors (including human, vehicle, physical and social environments) and phases (before, during, and after the accident) the Haddon’s matrix. Therefore, mobile apps can be considered as intervention tools in road traffic health and safety. Developers of mobile apps may think systematically about the comprehensive contents for mobile apps in road traffic health and safety mobile considering multiple factors presented in the Haddon matrix. Therefore, this could end up with more meaningful and effective mobile apps in this domain. Since road traffic accidents and injuries are multi-factorial issues, they require multi-factorial solutions.

## Supplementary Information

**Additional file 1**. Complete characteristics and features of Road Traffic apps retrieved from Google Play, and categorization of them by Haddon matrix.

## Data Availability

The datasets used and/or analyzed during the current study are available from the corresponding author on reasonable request.
